# Second-trimester dyadic coping and risk of third-trimester depressive symptoms: development and internal validation of a prediction model

**DOI:** 10.3389/fmed.2026.1892119

**Published:** 2026-08-17

**Authors:** Bingxin Wang, Yifan Jiang, Mengru Liu, Congcong Cao, Yan Peng, Yangyang Li, Yan Wang, Yuhan Cheng, Fang Fang, Yanping Zhuang

**Affiliations:** 1 Department of Nursing, Shanghai General Hospital, Shanghai Jiao Tong University School of Medicine, Shanghai, China; 2 School of Nursing, Bengbu Medical University, Bengbu, China; 3 School of Nursing, Shanghai Jiao Tong University, Shanghai, China; 4 Department of Nursing, Tongji Hospital Affiliated to Tongji University, Shanghai, China; 5 Department of Nursing, Shanghai Tenth People’s Hospital Affiliated to Tongji University, Shanghai, China

**Keywords:** antenatal depressive symptoms, dyadic coping, internal validation, pregnancy, risk prediction model, third trimester

## Abstract

**Background:**

Depressive symptoms in late pregnancy are common and may adversely affect maternal well-being and perinatal outcomes. Current antenatal screening mainly relies on symptom scales administered at a single time point, which may be insufficient for identifying women who are likely to develop depressive symptoms later in pregnancy. Dyadic coping reflects how partners communicate, support each other, and respond jointly to stress, but its value in risk prediction for third-trimester depressive symptoms remains unclear.

**Methods:**

This prospective observational study recruited pregnant women receiving routine antenatal care at a tertiary hospital in Shanghai, China. Recruitment lasted from January 2025 to August 2025. Baseline predictors were collected at 21–24 weeks of gestation and included demographic characteristics, pregnancy-related clinical data, laboratory indicators, and dyadic coping. Third-trimester depressive symptoms were assessed at 28 to 36 + 6 weeks of gestation using the 24-item Hamilton Depression Rating Scale. Multivariable logistic regression was used to develop a basic clinical-demographic model and a dyadic coping-enhanced model. Model performance was evaluated using discrimination, calibration, Brier score, decision curve analysis, and bootstrap internal validation.

**Results:**

A total of 370 complete cases were included, of whom 86 developed third-trimester depressive symptoms. During baseline screening, 11 women were excluded solely for meeting the exclusion criterion of baseline HAMD-24 total score ≥8. Women with depressive symptoms had significantly lower dyadic coping scores than those without symptoms. In the basic model, younger age, higher mean platelet volume, gestational hypertension, intrauterine growth and amniotic fluid abnormalities, and immune and autoimmune diseases were associated with third-trimester depressive symptoms. After adding dyadic coping, the area under the curve increased from 0.739 to 0.826, and the Brier score decreased from 0.152 to 0.127. The enhanced model also showed significant improvement in integrated discrimination improvement and net reclassification improvement. After bootstrap correction, the area under the curve was 0.719 for the basic model and 0.810 for the dyadic coping-enhanced model. Decision curve analysis showed that the enhanced model provided greater net benefit across most clinically relevant threshold probabilities.

**Conclusion:**

Dyadic coping assessed in the second trimester provided incremental predictive value for third-trimester depressive symptoms beyond routinely available clinical information. Incorporating dyadic coping into antenatal risk assessment may provide preliminary risk screening reference for our single-center cohort to identify women requiring enhanced psychological and family support. However, multi-center external validation across diverse obstetric populations is required before this model can be routinely implemented in general clinical settings.

## 1 Introduction

Antenatal depressive symptoms are among the most common mental health problems in pregnant women, with a reported prevalence of approximately 28.5%–42.7%, and represent an important risk condition requiring attention in perinatal care (1, 2). Depressive symptoms refer to a group of emotional, cognitive, behavioral, and physiological abnormalities, mainly including persistent low mood, loss of interest, and anhedonia. Antenatal depressive symptoms may reduce maternal quality of life, self-care ability, and adherence to prenatal care, and may also adversely affect maternal and infant outcomes through sleep disturbance, behavioral changes, neuroendocrine dysregulation, inflammatory responses, and impaired family functioning (3). Depressive symptoms in the third trimester deserve particular attention. From 28 weeks of gestation onwards, pregnant women often face increasing physical discomfort, poorer sleep quality, fear of childbirth, concerns about fetal health, and stress related to role transition, all of which may further increase psychological vulnerability (4). The clinical diagnosis of depression requires a strict diagnostic process conducted by psychiatrists, which is difficult to implement on a large scale in routine antenatal screening. In contrast, depressive symptoms can be rapidly identified using standardized clinician-administered scale, and early warning at the symptom level is more feasible for preventing disease progression and enabling timely intervention (5). Gestational weeks 21–24 represent a key antenatal visit window for systematic fetal ultrasound screening, during which hospital attendance and compliance are generally high. At the same time, rapid changes in maternal hormone levels may increase emotional fluctuations and susceptibility to negative emotions (6). Therefore, identifying women at high risk of third-trimester depressive symptoms during the second trimester, particularly at 21–24 weeks of gestation, has important clinical significance for timely psychological assessment, family support interventions, and referral when necessary.

At present, the clinical identification of antenatal depressive symptoms mainly relies on psychological screening scales administered at a single time point. Although these scales can detect existing depressive symptoms, they have limited ability to identify pregnant women who are at high risk of developing depressive symptoms later in pregnancy. Previous studies have shown that age, educational level, economic status, obstetric history, previous adverse pregnancy history, pregnancy complications, sleep quality, social support, and prior psychological status may all be associated with antenatal depressive symptoms (7, 8). However, existing studies on risk identification and prediction have mainly focused on individual-level demographic, obstetric, and general psychosocial factors, with insufficient attention paid to coping resources at the level of couple interaction and the family system.

Pregnancy is not only a physiological process for women, but also an important period during which couples jointly face role transition, changes in caregiving responsibilities, and stress related to preparation for parenthood. Dyadic coping refers to the process by which partners collaboratively adjust to stressful situations through stress communication, emotional support, problem-solving, and joint coping (9). Unlike general social support, dyadic coping emphasizes the quality of couple interaction and the ability to share responsibilities under stress. Better dyadic coping may help pregnant women obtain more stable emotional comfort, practical support, and a sense of shared responsibility, thereby reducing the negative impact of pregnancy-related stress on psychological status. Conversely, insufficient dyadic coping may increase loneliness, uncertainty, family conflict, and psychological burden, thereby increasing the risk of depressive symptoms in the third trimester (10).

Therefore, the value of dyadic coping in research on antenatal depressive symptoms should not be limited to whether it is associated with depressive symptoms. From a clinical perspective, a more relevant question is whether dyadic coping can further improve the identification of women at risk of third-trimester depressive symptoms beyond routine demographic and obstetric information. If dyadic coping provides incremental predictive value, it may serve not only as a psychosocial correlate, but also as an important basis for prenatal psychological risk screening and for identifying women who may benefit from couple-based supportive interventions. The second trimester is a suitable period for early psychological risk assessment and family system support, as most pregnant women have entered regular prenatal care while there remains sufficient time before the third trimester and delivery.

Accordingly, this study aimed to develop and internally validate a risk prediction model for third-trimester depressive symptoms by incorporating dyadic coping assessed at 21–24 weeks of gestation, together with routine demographic and obstetric information. In addition, we compared a conventional clinical-demographic model with an enhanced model that included dyadic coping, in order to evaluate the incremental value of dyadic coping in predicting third-trimester depressive symptoms. The findings may provide evidence for the early identification of prenatal psychological risk, stratified management of high-risk pregnant women, and the development of subsequent couple-based supportive interventions.

## 2 Methods

### 2.1 Study design and participants

This was a prospective observational study designed to develop and internally validate a risk prediction model for third-trimester depressive symptoms. Participants were pregnant women receiving routine antenatal care at the obstetric outpatient department of a tertiary hospital in Shanghai, China. Consecutive recruitment was carried out between January 2025 and August 25. Eligible women were consecutively recruited during the second trimester, and 21–24 weeks of gestation was defined as the baseline window for predictor assessment. All independent variables included in the prediction model were restricted to data that had been assessed, examined, or clearly recorded in the medical records during this window, including demographic characteristics, pregnancy-related clinical data, laboratory indicators, and dyadic coping. Pregnancy-related complications, clinical abnormalities, or other events that newly occurred or were first diagnosed after 24 weeks of gestation were not included as predictors in the model. For participants with two or more prenatal visits, questionnaire assessments, or laboratory tests within the 21–24-week window, the record closest to 24 weeks of gestation was used as the model input; if two records were equally close, the later record was selected. Depressive symptoms were assessed during follow-up in the third trimester, from 28 to 36 + 6 weeks of gestation.

### 2.2 Inclusion and exclusion criteria

#### 2.2.1 Inclusion criteria

Participants were eligible if they met all of the following criteria: (1) age ≥18 years; (2) singleton pregnancy; (3) gestational age of 21–24 weeks at baseline assessment; (4) ability to understand and complete the study questionnaires; (5) being in a stable partner relationship and able to complete the dyadic coping assessment; and (6) voluntary participation in the study and provision of written informed consent.

#### 2.2.2 Exclusion criteria

Participants were excluded if they met any of the following criteria: (1) a confirmed diagnosis of severe mental disorder, cognitive impairment, severe communication disorder, or any other condition that prevented completion of the questionnaire assessment before pregnancy; (2) severe pregnancy complications or critical conditions requiring emergency treatment at recruitment; (3) a baseline total score of ≥8 on the 24-item Hamilton Depression Rating Scale (HAMD-24); (4) confirmed severe fetal structural anomalies at baseline; (5) pregnancy termination, voluntary withdrawal, or loss to follow-up during the study; or (6) missing primary outcome data.

### 2.3 Sample size consideration

Sample size was determined following the criteria proposed by Riley et al. (11) for developing prediction models with binary outcomes. Based on clinical plausibility and prior literature, six predictor parameters were prespecified: five clinical-demographic variables (second-trimester age, mean platelet volume, gestational hypertension, intrauterine growth and amniotic fluid abnormalities, and immune and autoimmune diseases) and the dyadic coping total score. The basic model comprised the five clinical variables, while the enhanced model added dyadic coping as the sixth predictor. Considering the clinical significance of the 24-item Hamilton Depression Rating Scale (HAMD-24), this variable was further incorporated into both models. The following parameters were used in the pmsampsize package (R version 4.3.0 used for sample size calculation only): anticipated outcome prevalence of 23%, Cox-Snell R^2^ of 0.15, and a target shrinkage factor of 0.9. The minimum required sample size was 334 participants (77 outcome events). Accounting for a 10% attrition rate, we planned to enroll 370 pregnant women, expected to yield approximately 85 events, which exceeded the minimum requirement. The events-per-variable was 14.2 for the enhanced model, exceeding the recommended minimum of 10.

### 2.4 Data collection

Eligible women were screened and recruited during routine antenatal visits at 21–24 weeks of gestation. After written informed consent was obtained, uniformly trained research staff used standardized questionnaires to collect demographic characteristics, pregnancy-related information, baseline depressive symptoms, and dyadic coping. Relevant clinical diagnoses and laboratory indicators were verified through the hospital electronic medical record system. All candidate predictors were based on information available within the 21–24-week baseline predictor assessment window. For binary clinical diagnosis variables, a variable was coded as “yes” only if it had been clearly documented in the medical records or clinically diagnosed as present during this window. If the diagnosis first occurred or was first confirmed after 24 weeks of gestation, it was not treated as a positive baseline predictor and was considered absent at baseline. For participants with multiple antenatal visits, questionnaire assessments, or laboratory tests within the baseline window, the record closest to 24 weeks of gestation was selected; if two records were equally close, the later record was used. Baseline depressive symptoms were assessed using the HAMD-24 to exclude women with depressive symptoms at baseline. Third-trimester depressive symptoms were assessed again using the HAMD-24 during antenatal visits at 28 to 36 + 6 weeks of gestation.

### 2.5 Outcome definition

The primary outcome of this study was third-trimester depressive symptoms. Third-trimester depressive symptoms were assessed using the HAMD-24 (12). The scale contains 24 items. According to commonly used scoring criteria, a total HAMD-24 score of 0–7 indicates no depressive symptoms, whereas a score of ≥8 indicates the presence of depressive symptoms. Follow-up assessment in the third trimester was conducted at 28 to 36 + 6 weeks of gestation during routine antenatal visits. Each participant underwent one outcome assessment during this predefined window; the outcome was not assessed over a 3–12-month follow-up period. Therefore, the primary outcome was defined as third-trimester depressive symptoms, namely a total HAMD-24 score of ≥8 (13). The HAMD-24 rather than the EPDS was used as the outcome assessment tool because HAMD-24 is a clinician-administered, observer-rated scale that provides a more structured evaluation of depressive symptoms and reduces potential self-report bias. Although the EPDS is widely used for screening in perinatal populations, HAMD-24 was considered more appropriate for this study as it relies on standardized clinical assessment performed by trained raters, which is consistent with the design of this prediction model. The HAMD-24 assessments were conducted by two trained research nurses who were blinded to baseline characteristics and outcomes. All assessors received standardized training and used a unified scoring manual to ensure consistency in administration. Regular supervision and consensus discussions were conducted during the assessment period to minimize inter-rater variability; however, inter-rater reliability was not formally quantified in this study.

### 2.6 Candidate predictors

Candidate predictors were prespecified based on clinical plausibility, prior literature, and routine availability at the 21- to 24-week antenatal visit. For the basic clinical-demographic model, six predictors were selected *a priori*: baseline HAMD-24 score, maternal age, mean platelet volume, gestational hypertension, intrauterine growth and amniotic fluid abnormalities, and immune and autoimmune diseases. For the dyadic coping-enhanced model, the dyadic coping total score was added as a seventh predictor. Other demographic, obstetric, and laboratory variables were recorded for descriptive baseline comparisons (Table 1) but were not considered as candidate predictors in the multivariable models.

**Table 1:** Univariate analysis of baseline factors associated with third-trimester depressive symptoms.

Variable	Without depressive symptoms (*n* = 284)	With depressive symptoms (*n* = 86)	Statistic	*p* value
Dyadic coping score	127.70 ± 16.59	112.60 ± 14.10	7.647	<0.001
HAMD-24 score	3.44 ± 2.26	3.24 ± 2.37	0.685	0.494
Age	31.69 ± 4.04	30.67 ± 4.24	2.014	0.045
Hemoglobin level	115.84 ± 13.53	112.27 ± 14.48	2.110	0.036
Red blood cell count	3.73 ± 0.43	3.69 ± 0.39	0.808	0.420
Platelet count	194.78 ± 49.00	187.90 ± 42.68	1.173	0.242
White blood cell count	8.99 ± 2.23	8.97 ± 2.13	0.075	0.940
Neutrophil percentage	73.33 ± 6.11	73.49 ± 5.65	−0.216	0.829
Lymphocyte percentage	19.98 ± 5.31	19.27 ± 5.01	1.106	0.269
Monocyte percentage	5.44 ± 1.29	5.29 ± 1.25	0.913	0.362
Eosinophil percentage	1.54 ± 0.89	1.72 ± 1.06	−1.565	0.119
Basophil percentage	0.19 ± 0.12	0.21 ± 0.14	−0.827	0.409
Hematocrit	34.23 ± 3.89	33.74 ± 3.53	1.036	0.301
Mean corpuscular volume	91.91 ± 6.00	91.83 ± 4.76	0.122	0.903
Mean corpuscular hemoglobin	31.08 ± 2.13	31.09 ± 1.90	−0.058	0.954
Red cell distribution width	48.06 ± 4.22	48.96 ± 5.07	−1.659	0.098
Mean platelet volume	9.85 ± 1.12	10.33 ± 1.11	−3.481	<0.001
C-reactive protein	36.31 ± 23.75	39.18 ± 23.50	−0.985	0.325
Thyroid dysfunction [*n* (%)]			0.365	0.546
Yes	86 (30.3)	29 (33.7)		
No	198 (69.7)	57 (66.3)		
Anemia [*n* (%)]			0.052	0.819
Yes	21 (7.4)	7 (8.1)		
No	263 (92.6)	79 (91.9)		
Gynecological and reproductive system diseases [*n* (%)]			0.588	0.443
Yes	40 (14.1)	15 (17.4)		
No	244 (85.9)	71 (82.6)		
Infectious diseases [*n* (%)]			1.079	0.299
Yes	21 (7.4)	3 (3.5)		
No	263 (92.6)	83 (96.5)		
Endocrine and metabolic diseases [*n* (%)]			0.337	0.562
Yes	33 (11.6)	12 (14.0)		
No	251 (88.4)	74 (86.0)		
Hematological diseases [*n* (%)]			0.030	0.863
Yes	4 (1.4)	1 (1.2)		
No	280 (98.6)	85 (98.8)		
Gestational vascular and thrombotic disorders [*n* (%)]			0.007	0.933
Yes	3 (1.1)	1 (1.2)		
No	281 (98.9)	85 (98.8)		
Intrauterine growth and amniotic fluid abnormalities [*n* (%)]			6.437	0.011
Yes	25 (8.8)	16 (18.6)		
No	259 (91.2)	70 (81.4)		
Placental and umbilical cord abnormalities [*n* (%)]			0.056	0.813
Yes	22 (7.7)	6 (7.0)		
No	262 (92.3)	80 (93.0)		
Fetal distress [*n* (%)]			1.450	0.228
Yes	11 (3.9)	6(7.0)		
No	273 (96.1)	80 (93.0)		
Gestational diabetes mellitus [*n* (%)]			0.731	0.393
Yes	48 (16.9)	18 (20.9)		
No	236 (83.1)	68 (79.1)		
Immune and autoimmune diseases [*n* (%)]			5.762	0.016
Yes	31 (10.9)	18 (20.9)		
No	253 (89.1)	68 (79.1)		
Gestational hypertension [*n* (%)]			112.057	<0.001
Yes	26 (9.2)	54 (62.8)		
No	258 (90.8)	32 (37.2)		

#### 2.6.1 Sociodemographic characteristics

Maternal age at 21–24 weeks of gestation was included as the primary sociodemographic predictor.

#### 2.6.2 Pregnancy-related clinical data

Pregnancy-related clinical data were extracted from electronic medical records. The following variables were assessed at the 21–24-week baseline window.

Gestational hypertension was diagnosed based on standard clinical criteria (14), defined as systolic blood pressure ≥140 mmHg and/or diastolic blood pressure ≥90 mmHg first occurring after 20 weeks of gestation, with no prior history of hypertension. All cases of gestational hypertension included in the model were required to have been clearly documented in the medical records or clinically diagnosed within the 21–24-week baseline predictor assessment window. If the diagnosis first occurred or was first confirmed after 24 weeks of gestation, it was not considered a positive baseline predictor. All diagnoses were double-verified through the hospital electronic medical record system to ensure data accuracy.

*Intrauterine growth and amniotic fluid abnormalities*: This composite variable included the following sub-diagnoses confirmed by ultrasound or clinical assessment within the 21–24-week baseline window: (1) fetal growth restriction (estimated fetal weight or abdominal circumference below the 10th percentile for gestational age); (2) polyhydramnios (amniotic fluid index ≥24 cm or maximum vertical pocket ≥8 cm); and (3) oligohydramnios (amniotic fluid index ≤5 cm or maximum vertical pocket ≤2 cm).

*Immune and autoimmune diseases*: This composite variable included the following conditions with a confirmed diagnosis within the 21–24-week baseline window: (1) systemic lupus erythematosus; (2) antiphospholipid syndrome; (3) Hashimoto’s thyroiditis; (4) rheumatoid arthritis; and (5) other autoimmune diseases requiring immunomodulatory therapy.

Mean platelet volume (MPV) was measured as a continuous variable from routine blood tests conducted at 21–24 weeks of gestation.

#### 2.6.3 Dyadic coping

Dyadic coping was assessed using the Chinese version of the Dyadic Coping Inventory (DCI) (15), a 37-item self-report instrument that measures how couples communicate about stress and engage in joint coping. The DCI comprises five subscales: stress communication (8 items: 4 for self and 4 for partner), supportive dyadic coping (10 items: 5 for self and 5 for partner), common dyadic coping (5 items), negative dyadic coping (8 items, reverse-scored), and evaluation of dyadic coping (2 items). Each item is rated on a 5-point Likert scale from 1 (never) to 5 (very often). After reverse-coding the negative coping items, a total score is calculated by summing all 37 items, yielding a possible range of 37 to 185, with higher scores indicating better dyadic coping. The total score was used as a continuous predictor in the enhanced model. The Chinese version of the DCI has been validated and widely used in Chinese perinatal populations (15–17). In our study, the DCI was administered by trained research staff using standardized instructions during the 21- to 24-week antenatal visit.

#### 2.6.4 Laboratory parameters

Laboratory indicators were obtained from fasting venous blood tests conducted within the 21–24-week baseline predictor assessment window. For participants with two or more laboratory test records within this window, the result closest to 24 weeks of gestation was selected; if two results were equally close, the later result was used. The laboratory indicators included the following:

(1) Routine blood indicators: hemoglobin, red blood cell count, platelet count, white blood cell count, neutrophil percentage, lymphocyte percentage, monocyte percentage, eosinophil percentage, basophil percentage, hematocrit, mean corpuscular volume, mean corpuscular hemoglobin, red blood cell distribution width, and mean platelet volume.
(2) Inflammatory indicator: C-reactive protein.

### 2.7 Missing data handling

Before model development, the proportion of missing data for each candidate predictor was examined. The overall missing proportion was extremely low (<1.2%), with no variable exceeding 5% missingness, and the primary outcome variable (third-trimester depressive symptoms) had zero missing values. Given the negligible missing rate (only eight cases, representing 2.0% of the screened sample, were excluded due to missing predictor values), complete-case analysis was adopted as the primary analytical approach. Multiple imputation was not performed, as the minimal missing proportion was judged unlikely to introduce substantive bias or alter the core conclusions of the prediction models.

### 2.8 Model development

Third-trimester depressive symptoms were used as the dependent variable, and multivariable logistic regression was used to develop the prediction models. Candidate predictors were pre-specified based on clinical relevance and prior evidence. All independent variables entered into the models were restricted to information obtained within the 21–24-week baseline predictor assessment window. Clinical events that newly occurred or were first confirmed after 24 weeks of gestation were not included in the models. Two models were planned in this study.

1. First, a conventional clinical-demographic model was developed. This model included demographic and obstetric variables routinely available in second-trimester antenatal outpatient care and was used to represent the baseline predictive ability without additional assessment of dyadic coping.
2. Second, a dyadic coping-enhanced model was developed. The total dyadic coping score was added to the conventional clinical-demographic model to evaluate whether dyadic coping could further improve the prediction of third-trimester depressive symptoms beyond routine information.
3. Continuous variables were retained as continuous variables in principle. Restricted cubic splines were used to examine the linearity between continuous variables and the logit of the outcome; if obvious non-linearity was observed, variable transformation was considered. Categorical variables were entered into the models as dummy variables. Multicollinearity was assessed using variance inflation factors. If obvious collinearity was present between variables, they were not entered into the same model simultaneously.

### 2.9 Model performance evaluation

Model performance was evaluated in terms of discrimination, calibration, overall accuracy, and clinical utility. Discrimination was assessed using the area under the receiver operating characteristic curve (AUC), with 95% confidence intervals (CIs) reported. For clinically meaningful risk thresholds, sensitivity, specificity, positive predictive value, and negative predictive value were further reported.

Calibration was quantitatively evaluated via core metrics including calibration intercept, calibration slope (both reported with 95% confidence intervals [CIs]), and the Brier score alongside graphical calibration curves. A calibration slope close to 1 and a calibration intercept close to 0 reflect optimal concordance between predicted and observed event risks; overlapping of the calibration curve with the 45° reference line further visually confirms favorable calibration. The Hosmer–Lemeshow goodness-of-fit test was retained only as secondary auxiliary evidence and excluded from primary calibration judgment, given its well-documented limitation of strong sensitivity to sample size and arbitrary grouping thresholds, which reduces its reliabilityfor modern prediction model calibration assessment.

To quantify the incremental predictive value of adding dyadic coping to the base clinical-demographic model, integrated discrimination improvement (IDI) and net reclassification improvement (NRI) were calculated. The NRI metric adopted in this study was specified as continuous NRI (calculated across all predicted risk probabilities without predefined risk cutoffs), rather than categorical NRI based on manually divided risk strata. Due to inherent interpretive uncertainties and unstable performance of NRI across different risk distributions, IDI and decision curve analysis (DCA) were prioritized as the primary statistical evidence to judge incremental predictive gain, while continuous NRI only served as secondary supportive evidence.

Overall accuracy was assessed using the Brier score, with lower values indicating smaller overall prediction error.

Clinical utility was evaluated using decision curve analysis. By comparing the net benefit of the model across different threshold probabilities with strategies of classifying all women as high risk or classifying all women as low risk, we assessed whether the model had greater potential clinical value.

### 2.10 Internal validation

Bootstrap resampling with 1,000 replicates was performed for internal validation to quantify overfitting optimism. After refitting the model on each bootstrap sample, performance metrics were estimated in both resampled and original datasets to generate optimism-corrected discrimination and calibration statistics, including corrected AUC, corrected calibration slope, and corrected Brier score. Quantified calibration intercept and slope with 95% CIs for both the basic and enhanced models were extracted as supplementary calibration outcomes.

If obvious overfitting was present, a uniform shrinkage factor was calculated based on the bootstrap results and used to adjust the regression coefficients. The final model could then be used to establish a prediction equation based on the regression coefficients.

### 2.11 Incremental value of dyadic coping

To evaluate the contribution of dyadic coping, model performance was compared between the conventional clinical-demographic model and the dyadic coping-enhanced model. The comparison included changes in AUC, Brier score, calibration performance, and net benefit in decision curve analysis. When appropriate, the net reclassification improvement and integrated discrimination improvement were also reported as supplementary evidence, but these results were not used as the sole basis for judging the value of the model.

The core criterion for evaluating the value of dyadic coping in this study was not whether it reached statistical significance in multivariable regression, but whether its addition to the model improved discrimination, calibration, and clinical net benefit.

### 2.12 Statistical analysis

Continuous variables were presented as the mean ± standard deviation or median and interquartile range according to their distribution, whereas categorical variables were presented as frequencies and percentages. Baseline characteristics were descriptively compared according to the presence or absence of third-trimester depressive symptoms. Continuous variables were compared using the independent-samples *t* test or the Mann–Whitney *U* test, and categorical variables were compared using the *χ^2^* test or Fisher’s exact test.

All statistical analyses were performed using SPSS version 27.0. Logistic regression was used for model development. Receiver operating characteristic curves, calibration curves, bootstrap internal validation, and decision curve analysis were used to evaluate model performance. All tests were two-sided, and *p* < 0.05 was considered statistically significant.

### 2.13 Ethical considerations

This study was approved by the Ethics Committee of the participating hospital (Fast-track Ethics Approval No. Yuan Lun Kuai [2025] 835). All participants provided written informed consent before enrollment. During the study, participant data were anonymized, and all data were used only for the analyses of this study. For women who were found to have obvious depressive symptoms or risk of psychological crisis during follow-up, the researchers recommended further psychological assessment according to hospital procedures and referred them to specialist care when necessary.

## 3 Results

### 3.1 Univariate analysis

**Figure 1:**
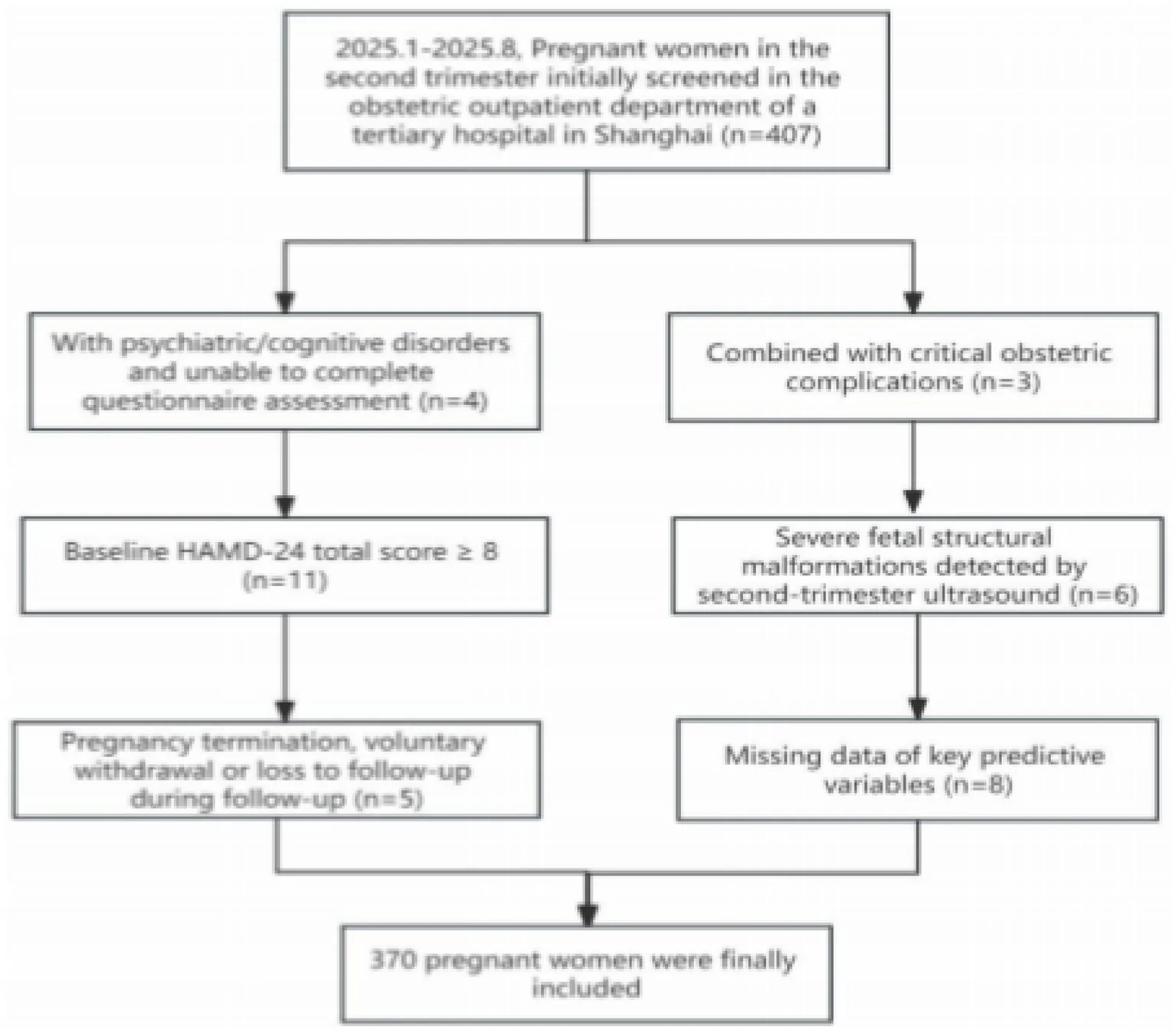
Patient selection flowchart.

This study initially recruited 407 women with second-trimester pregnancy who attended the obstetric outpatient clinic of a tertiary hospital in Shanghai from January 2025 to August 2025 as preliminary screening subjects. Screening was performed according to inclusion and exclusion criteria: 4 patients with mental and cognitive disorders who were unable to complete questionnaire assessments, 11 patients with a baseline 24-item Hamilton Depression Rating Scale (HAMD-24) score of ≥8 points, 5 patients who terminated their pregnancy, withdrew voluntarily or were lost to follow-up during the follow-up period; 3 patients complicated with severe critical pregnancy complications, 6 patients with severe fetal structural malformations indicated by second-trimester ultrasonography, and 8 patients with missing data of primary predictive variables, with a total of 37 cases excluded. Ultimately, 370 pregnant women meeting the study criteria were included, and the screening flow chart of study subjects is shown in Figure 1.

Univariate analysis was performed on the baseline demographic data, laboratory indicators, and pregnancy-related clinical variables of the 370 complete cases, grouped according to the presence or absence of depressive symptoms in the third trimester. The results showed that the dyadic coping score in the group with depressive symptoms was significantly lower than that in the group without (112.60 ± 14.10 vs. 127.70 ± 16.59, *p* < 0.001); Additionally, statistically significant differences between the two groups were observed in age during the second trimester, mean platelet volume, gestational hypertension, intrauterine growth abnormalities and amniotic fluid abnormalities, and immune dysregulation disorders (all *p* < 0.05), as shown in Table 1. Univariate comparisons between the outcome groups were performed solely for descriptive purposes to characterize the baseline sample (Table 1). These comparisons were not used to select or filter variables for the multivariable models. Based on the prespecified predictor sets described in Section 2.6, the six pre-defined clinical predictors entered into the basic model included baseline HAMD-24 score, second-trimester age, mean platelet volume, gestational hypertension, intrauterine growth and amniotic fluid abnormalities, and immune and autoimmune diseases. The enhanced model was constructed by adding the total dyadic coping score to all six predictors of the basic model, resulting in a total of seven predictors.

### 3.2 Multivariate logistic regression analysis

With the occurrence of third-trimester depressive symptoms as the dependent variable (absence = 0, presence = 1), all six prespecified clinical predictors (baseline HAMD-24 score, second-trimester age, mean platelet volume, gestational hypertension, intrauterine growth and amniotic fluid abnormalities, and immune and autoimmune diseases) were entered into a binary logistic regression model for the basic model. For the enhanced model, the dyadic coping total score was added as a seventh predictor. Continuous variables were entered as original values, and categorical variables were dummy coded. The coding scheme is shown in Table 2.

**Table 2:** Coding of independent variables.

Variable	Coding method
Age	Original value
Mean platelet volume	Original value
HAMD-24 score	Original value
Gestational hypertension	Yes = 1, No = 0
Intrauterine growth and amniotic fluid abnormalities	Yes = 1, No = 0
Immune and autoimmune diseases	Yes = 1, No = 0

The model results showed that increasing age was associated with a lower risk of third-trimester depressive symptoms (OR = 0.935, 95% CI: 0.876–0.999). In contrast, higher mean platelet volume (OR = 1.579, 95% CI: 1.238–2.014), gestational hypertension (OR = 4.089, 95% CI: 2.246–7.447), intrauterine growth and amniotic fluid abnormalities (OR = 3.554, 95% CI: 1.689–7.477), and immune and autoimmune diseases (OR = 2.745, 95% CI: 1.363–5.529) were associated with an increased risk of third-trimester depressive symptoms. Baseline HAMD-24 score was not independently associated with the outcome (OR = 0.947, 95% CI: 0.840–1.066, *p* = 0.366), as shown in Table 3.

**Table 3:** Multivariable logistic regression analysis of factors associated with third-trimester depressive symptoms.

Variable	Category	B	SE	Wald	*p*	OR	95% CI for OR	
							Lower	Upper
Age		−0.067	0.033	4.014	0.045	0.935	0.876	0.999
Mean platelet volume		0.457	0.124	13.547	<0.001	1.579	1.238	2.014
Gestational hypertension	Yes	1.408	0.306	21.206	<0.001	4.089	2.246	7.447
	No (reference)							
Immune and autoimmune diseases	Yes	1.010	0.357	7.984	0.005	2.745	1.363	5.529
	No (reference)							
Intrauterine growth and amniotic fluid abnormalities	Yes	1.268	0.380	11.161	0.001	3.554	1.689	7.477
	No (reference)							
Depression score		−0.055	0.061	0.818	0.366	0.947	0.840	1.066
Constant	—	−4.196	1.616	6.738	0.009	0.015	—	—

The regression equation for the basic model was as follows: Logit(P) = −4.196 − 0.067 × second-trimester age + 0.457 × second-trimester mean platelet volume + 1.408 × second-trimester gestational hypertension + 1.268 × second-trimester intrauterine growth and amniotic fluid abnormalities + 1.010 × second-trimester immune and autoimmune diseases − 0.055 × baseline HAMD-24 score.

### 3.3 Discriminative performance of the basic model

**Figure 2:**
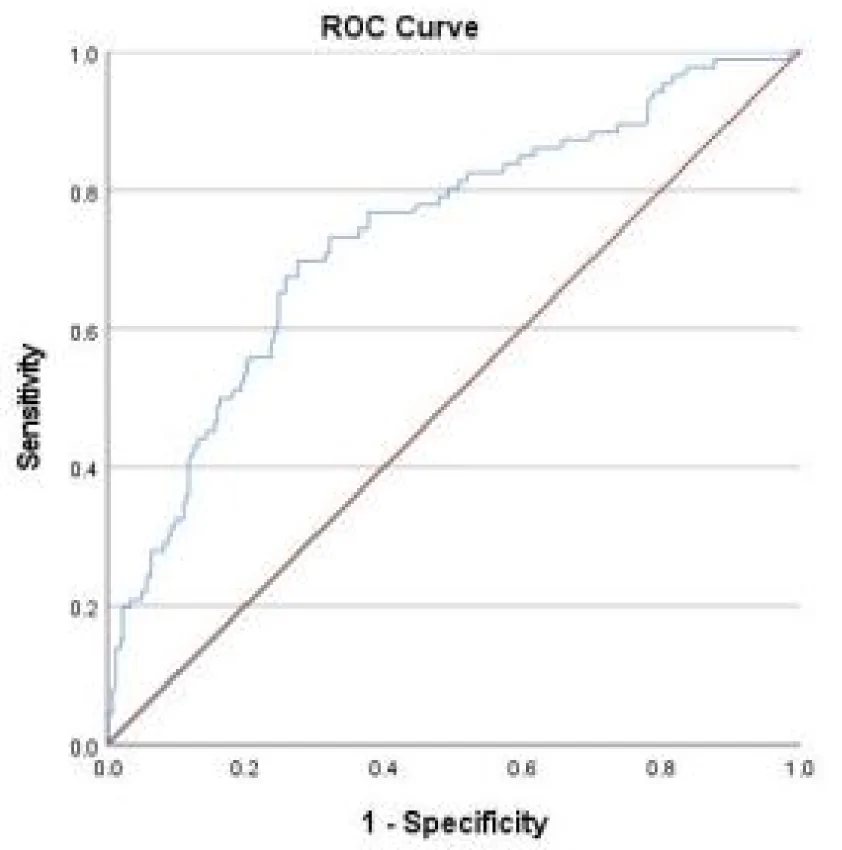
Receiver operating characteristic (ROC) curve of the basic logistic regression model.

A receiver operating characteristic (ROC) curve was constructed based on the predicted probabilities of the basic model. The results showed that the AUC of the basic logistic regression model was 0.739 (95% CI, 0.678–0.799), indicating that the model had moderate discriminative ability for third-trimester depressive symptoms, as shown in Figure 2.

**Figure 3:**
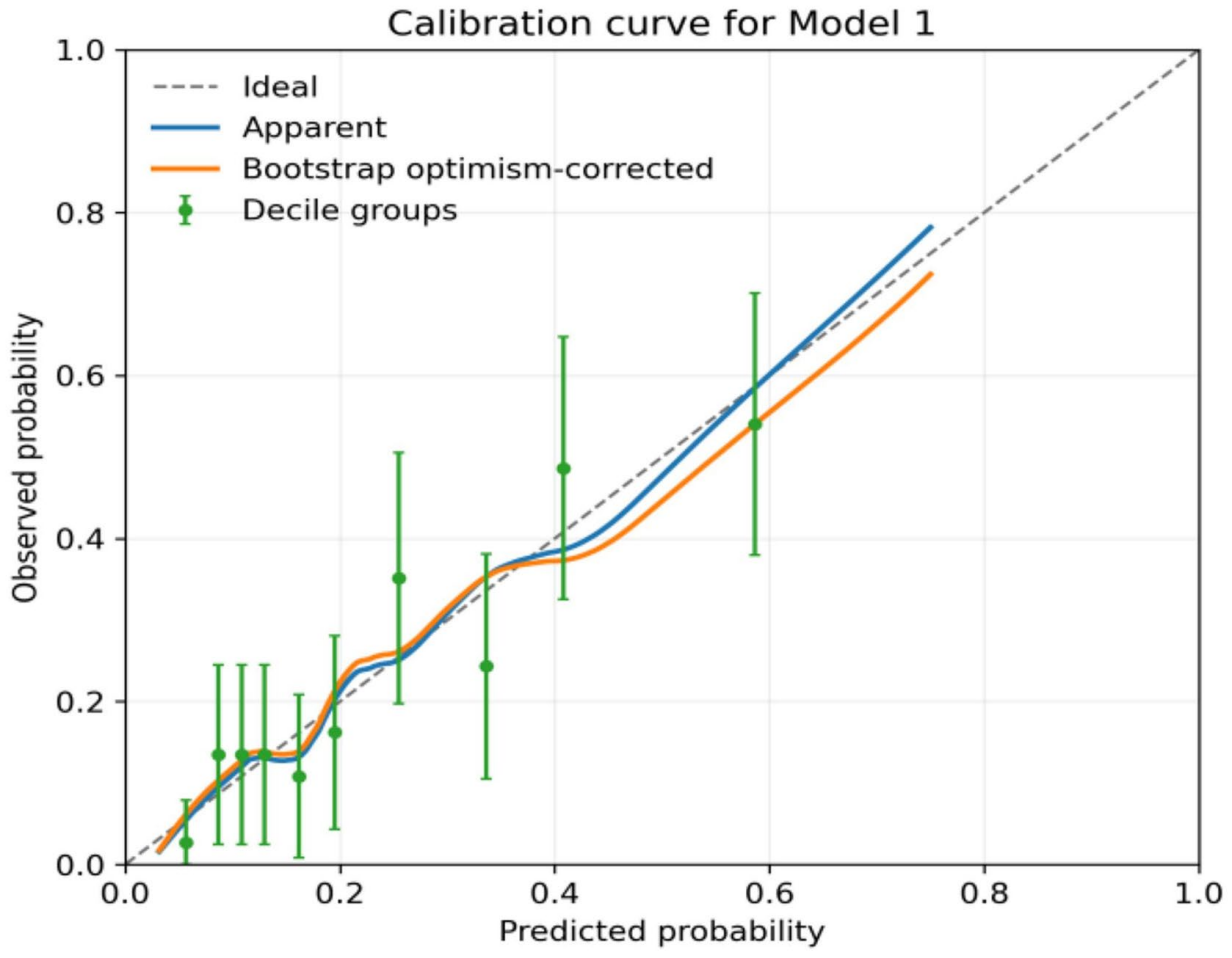
Calibration curve of the basic model.

Quantitative calibration metrics for the basic clinical-demographic model were extracted via bootstrap analysis: calibration intercept = 0.001 (95% CI, −0.255 ~ 0.285), calibration slope = 0.902 (95% CI, 0.676 ~ 1.206); with graphical calibration presented in Figure 3. The Hosmer–Lemeshow test for the basic model returned *p* = 0.480 consistent with the pre-specified analytical framework, this *p*-value was not used to judge overall model calibration due to the known sample-size-dependent bias of the test.

### 3.4 Comparison of predictive performance between the basic model and the dyadic coping-enhanced model

After the dyadic coping score was further added to the basic model, a dyadic coping-enhanced model was developed. The regression equation for the enhanced model was as follows: Logit(P) = 3.195–0.079 × age − 0.060 × dyadic coping score + 0.475 × mean platelet volume + 1.352 × gestational hypertension + 1.311 × intrauterine growth and amniotic fluid abnormalities + 0.913 × immune and autoimmune diseases − 0.054 × baseline HAMD-24 score.

**Figure 4:**
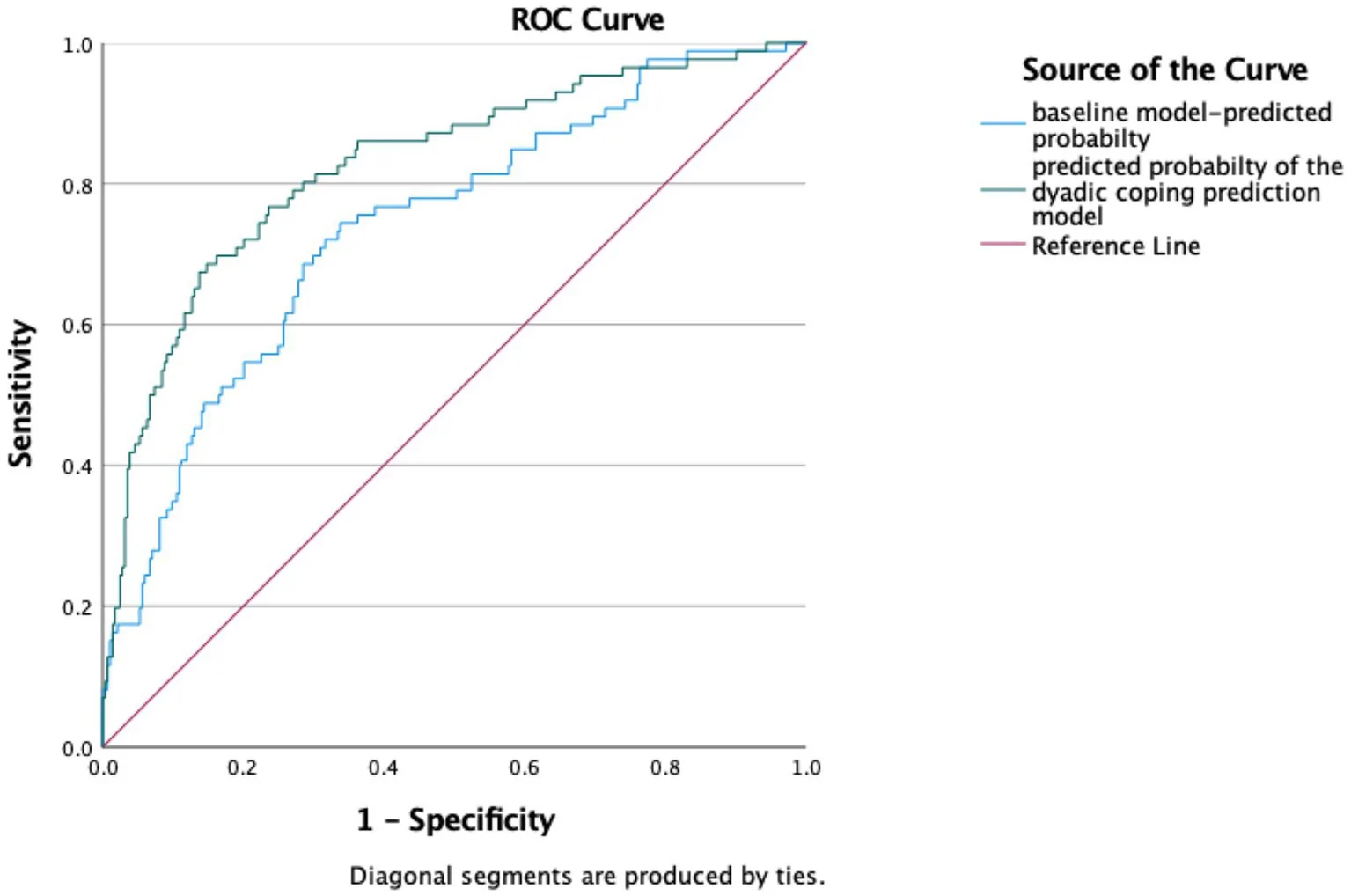
ROC curves of the two prediction models.

**Figure 5:**
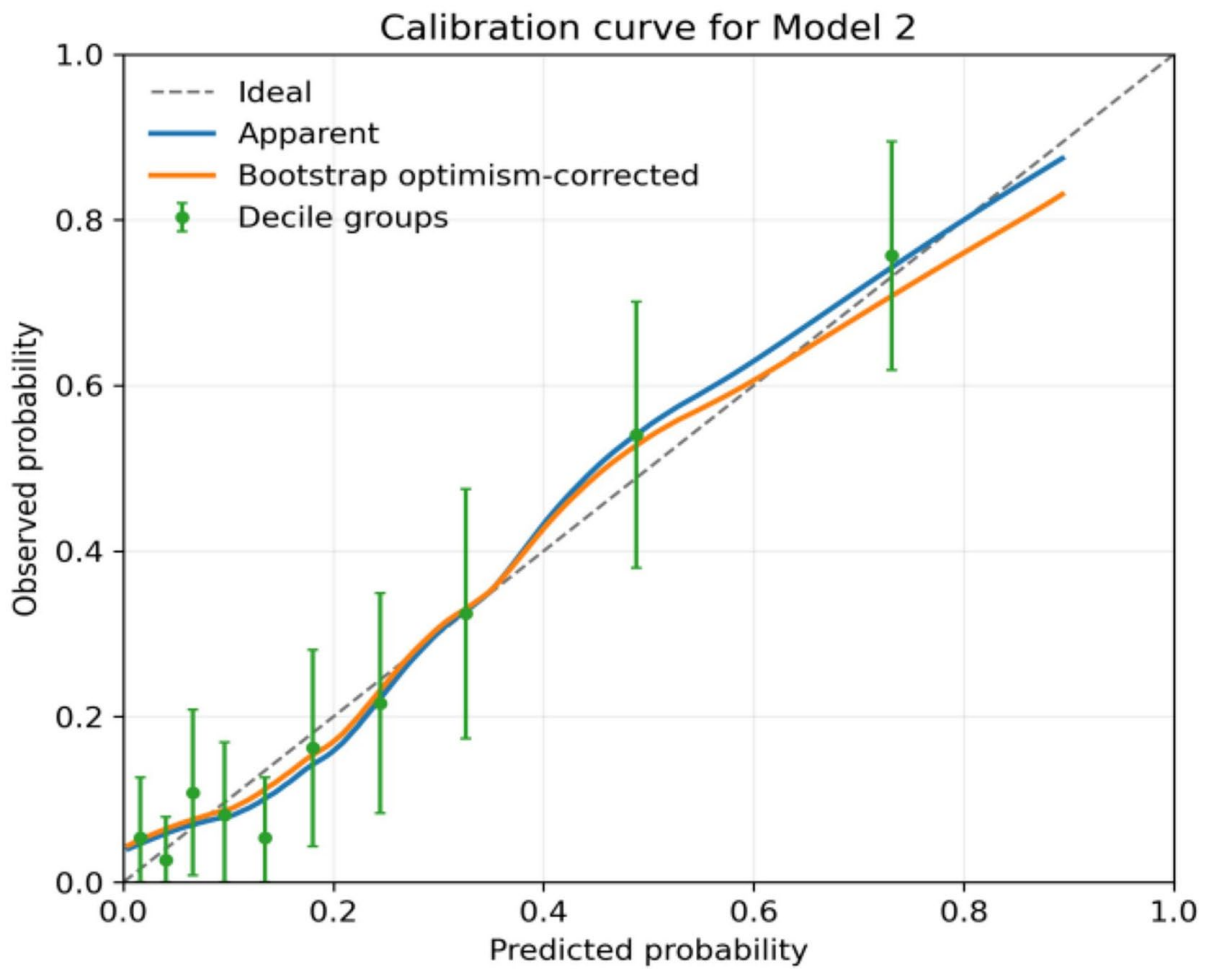
Calibration curve of the dyadic coping-enhanced model.

The dyadic coping-enhanced model had an AUC of 0.826 (95% CI: 0.770–0.876), which was higher than that of the basic model. At the optimal cut-off value of 0.3013, the sensitivity was 68.6%, the specificity was 85.2%, the positive predictive value was 58.4%, and the negative predictive value was 90.0%, as shown in Table 4 and Figure 4. The Hosmer–Lemeshow test yielded *χ*^2^ = 7.532 and *p* = 0.481; per analytical rules, this result was only treated as auxiliary calibration evidence rather than a primary calibration standard. Quantitative calibration metrics for the dyadic coping-enhanced model were as follows: calibration intercept = 0.001 (95% CI: −0.283~0.305), calibration slope = 0.911 (95% CI: 0.686~1.165). The near-ideal range of calibration slope and intercept, together with the graphical calibration curve (Figure 5), demonstrated strong concordance between model-predicted risks and observed prevalence of third-trimester depressive symptoms.

**Table 4:** Comparison of discriminative and diagnostic performance between the two prediction models for third-trimester depressive symptoms.

Variable	AUC (95% CI)	Hosmer-Lemeshow test *p* value	Cut-off value	Youden index	Sensitivity (95% CI)	Specificity(95% CI)	Positive predictive valueC (95% CI)	Negative predictive value (95% CI)
Model 1	0.739 (0.677 ~ 0.798)	0.480	0.1990	0.423	0.744 (0.643 ~ 0.825)	0.662 (0.605 ~ 0.715)	0.400 (0.327 ~ 0.477)	0.895 (0.846 ~ 0.930)
Model 2	0.826 (0.770~0.876)	0.481	0.3013	0.538	0.686 (0.582~0.774)	0.852 (0.806 ~ 0.889)	0.584 (0.487 ~ 0.675)	0.900 (0.858 ~ 0.930)

Relative to the basic clinical-demographic model, the Brier score of the dyadic coping-enhanced model declined from 0.152 to 0.127, reflecting a meaningful improvement in overall predictive accuracy. The enhanced model further revealed an IDI of 0.131 (95% CI: 0.071 to 0.211, *p* < 0.001) and a continuous NRI of 0.862 (95% CI: 0.574 to 1.072, *p* < 0.001). Consistent with the pre-specified weighting framework, the statistically significant positive IDI constituted the primary statistical evidence of dyadic coping’s incremental predictive utility, while the elevated continuous NRI value was interpreted only as supplementary supportive evidence due to well-documented limitations in NRI generalizability and interpretability. Collectively, these metrics demonstrated that integrating dyadic coping scores into routine second-trimester clinical data substantially improved model discrimination, risk reclassification performance, and overall predictive precision for incident third-trimester depressive symptoms.

### 3.5 Internal validation and clinical utility evaluation of the models

A total of 370 complete cases were included in this study, of whom 86 developed third-trimester depressive symptoms and 284 did not. Internal validation of the models was performed using bootstrap resampling with 1,000 resamples. The results showed that the apparent AUC of the basic model in the original sample was 0.739, and the optimism-corrected AUC was 0.719 (95% CI: 0.657 ~ 0.778). For the dyadic coping-enhanced model, the apparent AUC in the original sample was 0.826, and the optimism-corrected AUC was 0.810 (95% CI: 0.754 ~ 0.860). The corrected model performance showed only a slight decrease compared with the apparent performance, suggesting a low degree of overfitting and good internal stability, as shown in Figures 3, 5.

**Figure 6:**
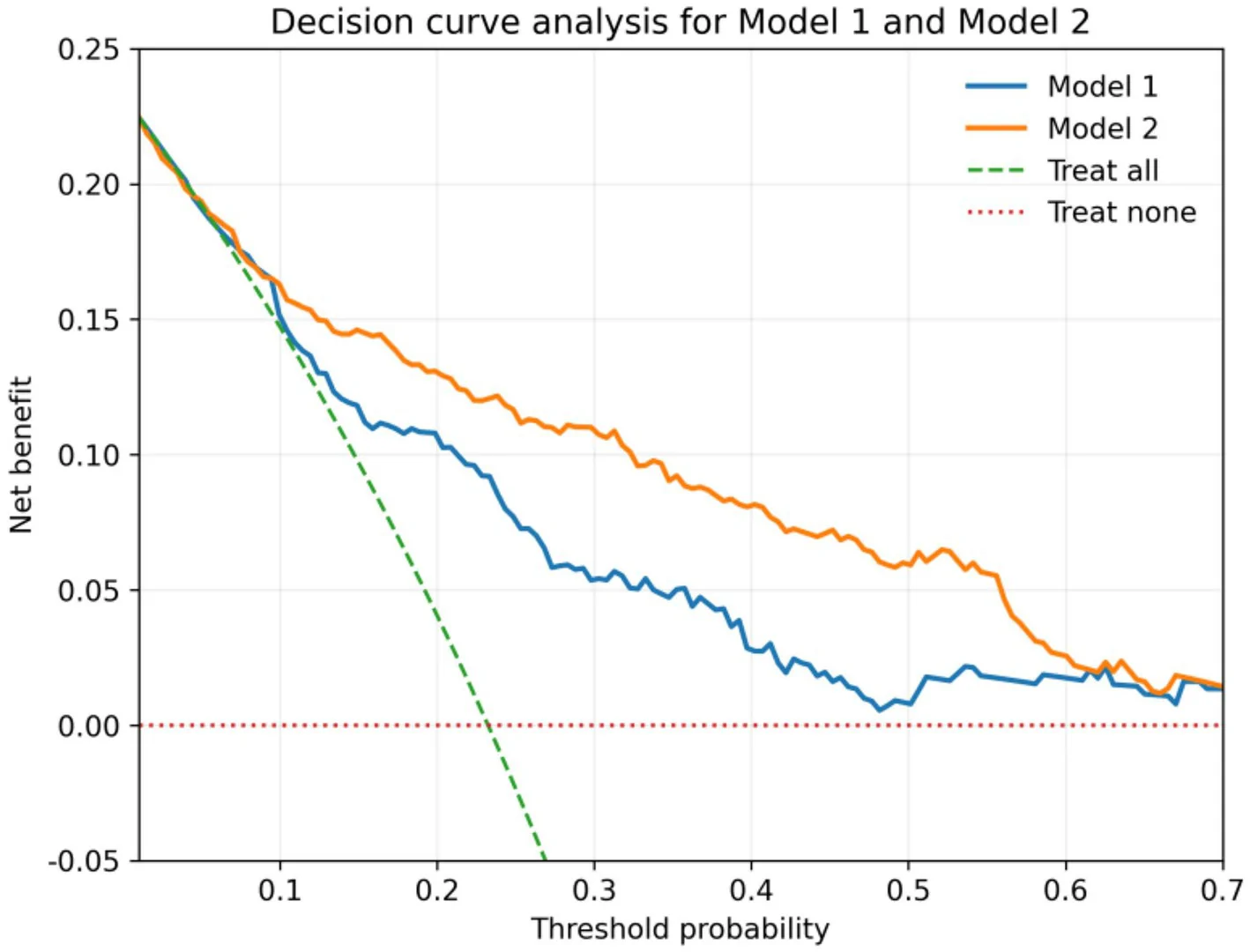
Decision curve analysis of the two prediction model.

Across threshold probabilities ranging from approximately 0.05 to 0.70, both models yielded higher net benefit than the reference strategies of treating all women as high-risk or treating no women as high-risk, which supports their clinical applicability. Within the narrower clinically meaningful threshold range of 0.01 to 0.61, the dyadic coping-enhanced model consistently delivered superior net benefit relative to the basic model, as illustrated in Figure 6.

This finding suggests that, in prenatal psychological risk screening where intervention costs are relatively low and the benefits of early identification are high, incorporating dyadic coping information may help identify pregnant women who require focused follow-up and support.

### 3.6 Sensitivity analyses

To assess whether the association between dyadic coping and third-trimester depressive symptoms was confounded by other clinically relevant variables, we incorporated gestational diabetes and thyroid dysfunction into the multivariable regression model simultaneously for sensitivity analysis. In the adjusted model, the odds ratio for dyadic coping remained 0.942 (*p* < 0.001). Gestational diabetes was not independently correlated with third-trimester depressive symptoms (OR = 1.061, 95% CI: 0.508–2.214, *p* = 0.875), and thyroid dysfunction also had no independent association with depressive symptoms (OR = 0.952, 95% CI: 0.515–1.763, *p* = 0.877). These results indicated that the predictive effect of dyadic coping was robust and not materially confounded by gestational diabetes or thyroid dysfunction.

## 4 Discussion

### 4.1 Incremental predictive value of dyadic coping: from individual risk to dyadic system risk

This study showed that second-trimester dyadic coping scores were significantly lower among women who developed depressive symptoms in the third trimester. Incorporating a single novel predictor (dyadic coping total score) into the base clinical-demographic model yielded a substantial AUC increment from 0.739 to 0.826, alongside a reduced Brier score (0.152 to 0.127) and statistically significant improvements in IDI and continuous NRI. It is important to contextualize the magnitude of this AUC gain: within standard prediction modeling benchmarks, an AUC increase of nearly 0.087 from one single predictor represents a comparatively large improvement in discriminative capacity. Notably, this performance advantage was only confirmed via bootstrap internal validation within a single-center cohort; consistent with prediction modeling reporting standards, external validation across independent obstetric populations is mandatory before this enhanced model can be formally adopted for routine clinical risk screening.

Per the pre-specified analytical weighting strategy, the robust positive IDI served as the core evidence of dyadic coping’s incremental predictive utility, rather than the elevated continuous NRI value, as NRI metrics are prone to variable interpretation across different risk distributions. Taken together, these findings suggest that dyadic coping is not merely a cross-sectionally correlated psychosocial correlate of depressive symptoms, but delivers independent, actionable risk stratification information unaccounted for by standard antenatal demographic, laboratory, and obstetric clinical data.

In other words, the occurrence of third-trimester depressive symptoms cannot be fully understood only from individual demographic characteristics, obstetric complications, or laboratory indicators. Relational factors, including the quality of couple interaction, joint coping ability, and the family support structure, should also be considered. Consistent with the systematic review by Pilkington et al. on modifiable partner-related factors, intimate relationship quality, communication, emotional support, instrumental support, and conflict were associated with the risk of perinatal depression and anxiety. Ding et al. also found in a Chinese sample of couples affected by gestational diabetes mellitus that negative dyadic coping mediated the association between perceived stress and antenatal depressive symptoms (16, 18). By placing dyadic coping within a prediction model framework, the present study further demonstrates that it can improve risk stratification for subsequent third-trimester depressive symptoms, thereby extending the clinical relevance of previous correlational findings.

Compared with previous studies, the contribution of this study is mainly reflected in three aspects. First, previous research on partner-related factors and perinatal mental health has mostly focused on cross-sectional associations or mediating mechanisms, addressing whether dyadic coping is associated with depressive symptoms. In contrast, this study approached the issue from the perspective of prospective risk identification and examined whether dyadic coping could improve the prediction of third-trimester depressive symptoms beyond conventional clinical data. Second, previous studies have often treated partner support, marital quality, or relationship conflict as general psychosocial variables, whereas this study emphasizes dyadic coping as a process of joint adjustment between partners under stress. It reflects whether pregnant women receive timely, stable, and practical partner responses when facing pregnancy-related stress. Third, this study incorporated dyadic coping into the model together with clinical variables such as age, mean platelet volume, gestational hypertension, intrauterine growth and amniotic fluid abnormalities, and immune and autoimmune diseases. This suggests that psychosocial resources and biomedical risks are not two separate categories of factors, but may jointly form a comprehensive risk structure for third-trimester depressive symptoms. Therefore, the significance of dyadic coping lies not only in explaining the psychosocial origins of depressive symptoms, but also in helping antenatal care shift from identifying only “clinically high-risk pregnant women” to identifying pregnant women whose clinical risks are compounded by insufficient family coping resources.

From a mechanistic perspective, stress in the middle and late stages of pregnancy is not borne solely by the pregnant woman herself. Instead, it is shaped through couple communication, responsibility sharing, emotional responsiveness, and joint problem-solving, forming a type of family-system stress (17). Physical discomfort, concerns about fetal health, the burden of antenatal examinations, fear of childbirth, and stress related to role transition may all be buffered through couple interaction. Conversely, they may be amplified by insufficient communication, avoidance, blame, or lack of support. Higher levels of dyadic coping may reduce the risk of depressive symptoms through three pathways. First, effective stress communication may help pregnant women express concerns about physical changes, fetal health, and uncertainty around childbirth, thereby reducing catastrophic interpretations and repeated worry caused by bearing stress alone. Second, emotional and instrumental support from partners may buffer the persistent stress caused by poor sleep, frequent antenatal visits, changes in daily routines, and adjustments in family responsibilities, allowing pregnant women to receive more direct help with specific problems. Third, joint coping may enhance pregnant women’s sense of control, being understood, and shared responsibility regarding pregnancy-related events, thereby reducing isolation, helplessness, and relationship conflict. Conversely, low dyadic coping may transform pregnancy-related stress within the couple into silence, avoidance, blame, or emotional withdrawal. As a result, pregnant women may have to face not only the uncertainty of pregnancy itself, but also relational stress caused by insufficient partner responsiveness, which may increase their psychological vulnerability to depressive symptoms.

In addition, dyadic coping may influence the risk of third-trimester depressive symptoms through psychological–biological pathways. Long-term relational stress and insufficient support may keep pregnant women in a state of sustained vigilance and chronic stress, thereby affecting hypothalamic–pituitary–adrenal axis activity, sleep rhythm, inflammatory responses, and autonomic nervous regulation (19). For pregnant women with existing clinical risks such as gestational hypertension, intrauterine growth and amniotic fluid abnormalities, or immune abnormalities, insufficient partner support may further weaken disease coping ability and treatment adherence, and may amplify concerns about fetal safety, delivery outcomes, and maternal health. In contrast, better dyadic coping may help pregnant women transform clinical risks into issues that can be discussed, shared, and managed. This may allow them to obtain more stable emotional support and practical assistance when facing abnormal test results, increased frequency of antenatal visits, or adjustments in treatment recommendations. Therefore, dyadic coping should not be viewed as a “soft” psychological factor independent of clinical risk. Rather, it may serve as an important regulatory resource that influences how pregnant women perceive, tolerate, and cope with clinical risk. This may also explain why adding dyadic coping to conventional clinical variables improved model discrimination, overall prediction error, and risk reclassification.

From the perspective of clinical translation, incorporating dyadic coping into second-trimester risk assessment may help shift the focus of prenatal psychological screening from individual disease risk to dyadic system risk. If assessment relies only on age, obstetric complications, and laboratory indicators, some pregnant women without prominent clinical risks may be underestimated. In contrast, women with low dyadic coping may still develop depressive symptoms in the third trimester because of insufficient family support, poor stress communication, and weak joint coping ability, even in the absence of obvious obstetric abnormalities. Compared with simply asking whether a pregnant woman feels anxious or depressed, dyadic coping assessment can further identify whether her stress is understood by her partner, whether the couple has the ability to solve problems together, and whether a stable support structure exists within the family. For high-risk pregnant women identified by the model, obstetric outpatient clinics may provide further psychological scale-based rescreening, couple-based health education, stress communication guidance, family support interventions, and referral to mental health specialists. In this way, risk prediction can move beyond the statistical model itself and be translated into a more specific pathway for stratified management.

However, reverse causation and construct overlap warrant caution. Although predictors were assessed before outcomes, subclinical depressive symptoms at baseline may still affect dyadic coping reports. We excluded women with HAMD-24 ≥8, but residual confounding remains possible. Additionally, baseline HAMD-24 as a predictor and outcome both derive from the same scale, introducing conceptual overlap; yet this does not compromise dyadic coping’s incremental value, as dyadic coping is conceptually distinct from depressive symptoms.

### 4.2 Integrated mechanisms of obstetric complications, platelet activation, and immune abnormalities

In this study, gestational hypertension, intrauterine growth and amniotic fluid abnormalities, immune and autoimmune diseases, and increased mean platelet volume were all associated with a higher risk of third-trimester depressive symptoms. This finding is consistent with the observation by Shay et al. that hypertensive disorders of pregnancy are associated with an increased risk of antenatal depressive and anxiety symptoms. It also echoes the findings of Chen et al. regarding the association between trajectories of depressive symptoms during pregnancy and adverse pregnancy outcomes (20, 21). However, previous studies have mostly focused on the effects of psychological symptoms on obstetric outcomes or on the comorbidity between the two. In contrast, the present study used clinically available indicators from the second trimester and suggests that a high-risk obstetric status itself may serve as an early signal of increased psychological risk in the third trimester (22). In other words, antenatal depressive symptoms may not be caused only by individual psychological vulnerability or insufficient social support. Clinical information, including obstetric risk, inflammatory status, vascular endothelial dysfunction, and immune imbalance, may also contribute to risk formation (23).

Gestational hypertension was one of the obstetric variables most strongly associated with third-trimester depressive symptoms in this study. Its influence may have both psychological and biological bases. From a psychological perspective, gestational hypertension often means more frequent antenatal visits, closer blood pressure monitoring, a higher likelihood of medication use or hospitalization, and persistent concerns about preterm birth, preeclampsia, placental dysfunction, and fetal safety (24). These clinical risks may place pregnant women in a state of sustained vigilance and increase catastrophic thinking and negative expectations. Unlike general pregnancy-related discomfort, gestational hypertension is often perceived by pregnant women as a clear medical risk that may threaten maternal and infant safety, and may therefore be more likely to trigger anxiety, helplessness, and low mood (25). From a biological perspective, hypertensive disorders of pregnancy are often accompanied by vascular endothelial dysfunction, placental hypoperfusion, oxidative stress, and increased inflammatory responses. These processes may overlap with hypothalamic–pituitary–adrenal axis activation, immune-inflammatory pathways, and abnormalities in neurotransmitter metabolism involved in depressive symptoms (26). Therefore, the association between gestational hypertension and third-trimester depressive symptoms may not be explained simply by “worry about the disease,” but may reflect the combined effects of high-risk obstetric status, chronic stress responses, and inflammatory vascular mechanisms. However, the relatively large effect estimate observed in this study should be interpreted cautiously. Gestational hypertension may represent a broader marker of high-risk pregnancy, greater medical burden, and increased concern about adverse maternal and fetal outcomes, rather than a direct causal factor for depressive symptoms. Residual confounding from unmeasured factors and the single-center study design may also have contributed to the strength of this association, which requires confirmation in larger multicenter cohorts.

The association between intrauterine growth and amniotic fluid abnormalities and an increased risk of third-trimester depressive symptoms is also clinically plausible. Abnormalities such as fetal growth restriction, polyhydramnios, or oligohydramnios usually directly point to concerns about fetal intrauterine safety and placental function (27). Compared with many maternal diseases, these abnormalities may more readily trigger concerns about fetal health, timing of delivery, neonatal prognosis, and the burden of family care. In particular, after such abnormalities are detected in the second trimester, pregnant women often require more intensive ultrasound monitoring, fetal assessment, and obstetric follow-up. Their psychological stress may not end after a single examination, but may continue to accumulate throughout the third trimester. In addition, intrauterine growth and amniotic fluid abnormalities may reflect placental perfusion problems, maternal–fetal circulatory abnormalities, inflammatory responses, or endocrine dysregulation. These biological changes may share common pathways with the development of emotional symptoms (28). Therefore, the findings of this study suggest that fetal-related abnormalities are not only important targets for monitoring obstetric outcomes, but may also serve as clinical signals of increased psychological risk in pregnant women.

Increased mean platelet volume was a laboratory indicator of interest in this study. Mean platelet volume is generally considered to be associated with the degree of platelet activation, and higher levels may indicate enhanced platelet reactivity, active coagulation–inflammation interactions, and microvascular dysfunction (29). Pregnancy itself is a relatively hypercoagulable state. When platelet activation, endothelial injury, and inflammatory responses are further enhanced, they may be linked to gestational hypertension, placental dysfunction, and other maternal–fetal circulatory abnormalities (30). In addition, platelets are involved not only in coagulation, but also in the release of inflammatory mediators, immune regulation, and serotonin metabolism. Depressive symptoms are thought to be related to inflammatory activation, neuroendocrine dysregulation, and changes in monoamine neurotransmitters. Therefore, increased mean platelet volume may not be an isolated hematological phenomenon, but rather a peripheral indicator reflecting combined abnormalities in vascular endothelial function, inflammatory status, and neuroimmune regulation (31). Including this indicator in the model may help capture biological risk information that cannot be reflected by demographic characteristics or obstetric diagnoses alone.

The association between immune and autoimmune diseases and an increased risk of third-trimester depressive symptoms can also be explained from several perspectives. These diseases often mean more complex disease management during pregnancy, more frequent follow-up monitoring, concerns about medication safety, and uncertainty regarding maternal and fetal outcomes. For pregnant women, immune-related diseases may bring not only physical symptoms and treatment burden, but also worries about disease recurrence, fetal development, delivery risks, and postpartum care. From a biological perspective, immune abnormalities may increase susceptibility to depressive symptoms through elevated pro-inflammatory cytokines, immune–neuroendocrine interactions, fatigue and pain burden, impaired sleep, and increased stress sensitivity (32). The inflammatory mechanisms of perinatal depression summarized by Zhu et al. also suggest that inflammatory cytokines, the tryptophan–kynurenine pathway, hypothalamic–pituitary–adrenal axis activation, and altered neurotransmitter metabolism may jointly contribute to the development of depressive symptoms (33). Therefore, the predictive value of immune and autoimmune diseases in this study may arise both from the psychological burden associated with a high-risk clinical condition and from underlying inflammatory and neuroimmune mechanisms.

Taken together, gestational hypertension, intrauterine growth and amniotic fluid abnormalities, increased mean platelet volume, and immune and autoimmune diseases should not be understood as isolated risk factors. Rather, they may collectively point to a broader risk chain involving high-risk pregnancy status, vascular endothelial dysfunction, inflammatory or immune activation, and increased psychological stress. Within this risk chain, obstetric complications increase pregnant women’s concerns about maternal and infant safety; platelet activation and immune abnormalities may reflect underlying vascular inflammation and neuroimmune imbalance; and continuous medical monitoring, risk communication, and uncertainty may further amplify psychological distress. These findings suggest that risk identification for third-trimester depressive symptoms should not rely only on traditional psychosocial factors, but should also consider obstetric and laboratory indicators that are already available in the second trimester. Incorporating these clinical variables together with family-system factors such as dyadic coping into a prediction model may help identify pregnant women with both biomedical risk and psychosocial vulnerability, providing a basis for subsequent stratified follow-up, psychological assessment, and integrated obstetric–psychological management.

### 4.3 Implications for antenatal clinical practice and future research

Notably, all findings of this single-center internally validated model cannot be generalized to other clinical settings, and routine clinical use is not recommended without external validation. The clinical significance of this study lies in moving the risk identification of third-trimester depressive symptoms forward to 21–24 weeks of gestation, a routine antenatal care window. This stage usually coincides with systematic ultrasound screening and several laboratory tests, with high attendance among pregnant women and convenient access to clinical data. It also leaves a meaningful time window for intervention before the third trimester and delivery. Compared with detecting obvious depressive symptoms only in the third trimester, identifying high-risk women in the second trimester may help obstetric teams conduct psychological rescreening, sleep assessment, partner communication guidance, and family support interventions earlier, thereby shifting risk management from passive detection to proactive early warning. For antenatal outpatient clinics, an ideal risk screening tool should not substantially increase the workload of healthcare providers or rely on indicators that are complex, expensive, or difficult to obtain repeatedly. The indicators included in the present model, including age, mean platelet volume, gestational hypertension, intrauterine growth and amniotic fluid abnormalities, and immune and autoimmune diseases, can be obtained from routine antenatal examinations and medical records. Dyadic coping can also be assessed using a questionnaire. This model only has preliminary research value for our local cohort and cannot be widely applied clinically without multi-center verification.

From the perspective of screening strategy, the findings of this study suggest that identifying the risk of third-trimester depressive symptoms should not rely only on whether pregnant women already show obvious low mood, loss of interest, or anxiety. Traditional antenatal psychological screening is mainly based on symptom scales, which can identify women who already have depressive symptoms. However, it may be insufficient for identifying women who have not yet reached the symptom threshold but may become high risk in the third trimester. The model developed in this study is closer to a pre-emptive risk stratification tool. It does not replace psychiatric diagnosis or standardized psychological scale screening. Instead, it uses clinical information and family-system information in the second trimester to identify women who require focused follow-up before obvious symptoms emerge. For women identified as high risk by the model, antenatal outpatient clinics may arrange further rescreening using the HAMD, the Edinburgh Postnatal Depression Scale, or other standardized psychological scales, together with comprehensive assessment of sleep quality, fear of childbirth, social support, and previous psychological status. For women with persistently high risk or clear signs of psychological crisis, psychological counselling or referral to mental health specialists may be initiated.

The decision curve analysis in this study showed that the dyadic coping-enhanced model had a higher net benefit in the low-threshold probability range, which is particularly important for antenatal psychological screening. The goal of antenatal psychological risk management is usually not to make an immediate psychiatric diagnosis or to provide high-intensity interventions for all pregnant women. Rather, it is to identify women who need continued attention at an earlier stage, when the risk is still relatively low and the cost of intervention is manageable. A higher net benefit in the low-threshold probability range suggests that when clinical teams are willing to initiate rescreening, follow-up, or health education at a relatively low risk threshold, the dyadic coping-enhanced model can identify more women who may truly develop third-trimester depressive symptoms while reducing unnecessary over-intervention. In other words, this model is more suitable for initial screening and stratified management in antenatal outpatient care than for use as a single diagnostic tool. Its main application should be to help healthcare providers decide who needs closer follow-up, who may benefit from partner involvement, and who requires further psychological specialist assessment.

In clinical practice, this model could be translated into a stratified management pathway. For low-risk pregnant women, routine antenatal care and general mental health education could be continued. For moderate-risk women, the frequency of psychological scale rescreening in the third trimester could be increased, and education on sleep, stress, and childbirth preparation could be strengthened. For high-risk women, a focused follow-up file could be established, with couple-based health education, stress communication guidance, family support interventions, and referral to psychological or psychiatric specialists when necessary. In particular, for women with low dyadic coping, the focus of intervention should not remain only on reminding the woman to regulate her emotions. Instead, the partner’s role should be incorporated, with specific guidance on stress expression, emotional responsiveness, attendance at antenatal visits, sharing of household responsibilities, childbirth preparation, and postpartum care arrangements. In this way, dyadic coping can be transformed from a predictive variable into a clinically actionable and manageable intervention target.

From the perspective of obstetric nursing, this model also provides an operational basis for nurse-led psychological risk management during pregnancy. Nurses have frequent contact with pregnant women in antenatal clinics, antenatal education programs, and follow-up management, and are well positioned to undertake initial risk screening, questionnaire assessment, health education, and stratified follow-up. Incorporating dyadic coping assessment into routine antenatal health education may help nurses identify women whose antenatal examination results appear acceptable, but who have insufficient partner support, poor communication quality, or weak family coping capacity. For these women, nursing interventions can move beyond simple knowledge education toward a couple-based support model. For example, nurses may guide partners to recognize stress signals in pregnant women, participate in communication and decision-making around antenatal care, jointly formulate childbirth preparation plans, and strengthen dynamic monitoring of sleep, mood fluctuations, and family conflict during the third trimester.

Future research may further focus on model translation and intervention application. First, the model developed in this study could be transformed into a simplified scoring tool, an automated alert module in the electronic medical record system, or an outpatient questionnaire screening form for preliminary research use only—formal clinical deployment is prohibited until rigorous multi-center external validation is fully completed. Second, independent external validation across geographically distinct hospitals, varying tiers of medical institutions, and diverse pregnant patient cohorts is a non-negotiable prerequisite before clinical application. This requirement is particularly salient given the unusually large AUC increment (0.087) observed after adding only dyadic coping as a single predictor; independent external testing is required to exclude overoptimism limited to the present single-center sample and confirm consistent discriminative and calibrated performance in external populations. Third, decision impact studies could be conducted to determine whether embedding the model into antenatal outpatient services improves psychological rescreening rates, referral rates, partner participation, and follow-up completion among high-risk women. Finally, as dyadic coping is a modifiable factor, couple-based psychological support interventions could be designed and evaluated for their effects on third-trimester depressive symptoms, antenatal anxiety, sleep quality, fear of childbirth, and postpartum adjustment. This would help move prediction models from risk identification toward proactive intervention.

## 5 Limitations

First, this single-center study only completed bootstrap internal validation without external cohort verification, which limits the generalizability of our model to other hospitals and pregnant populations. Clinical application is not recommended until multi-center external validation is performed. Several limitations of this study should be acknowledged. First, this was a single-center prospective observational study, and the model was only internally validated using bootstrap resampling. External validation in different regions, hospitals at different levels, and larger samples is still required. The relatively limited sample size may reduce the stability of regression coefficient estimates and weaken the reliability of model discrimination and calibration results. Second, the outcome was defined as third-trimester depressive symptoms assessed using the HAMD-24, which is not equivalent to a clinical diagnosis of depressive disorder. Since the prospective design supports temporal inference, reverse causation cannot be fully excluded, and using HAMD-24 both as a predictor and outcome creates construct overlap; however, this does not affect the interpretation of dyadic coping’s incremental value given its conceptual distinctness. Future studies could incorporate structured clinical interviews to improve the accuracy of outcome ascertainment. In addition, variation in the exact gestational week of outcome assessment within the predefined 28 to 36 + 6-week window may have introduced some temporal heterogeneity in depressive symptom measurement. Third, the model variables were mainly derived from a single time window at 21–24 weeks of gestation, and dynamic changes in dyadic coping, sleep quality, social support, and inflammatory markers were not incorporated. Fourth, although this study demonstrated the incremental predictive value of dyadic coping, its benefits as an intervention target need to be further verified in future intervention studies. Fifth, the enhanced model exhibited a large AUC improvement (0.739 to 0.826) following inclusion of a single psychosocial predictor (dyadic coping). All observed performance gains were only verified via bootstrap internal resampling within a single-center sample, without external cohort validation. The substantial magnitude of discriminative improvement raises concerns regarding generalizability to other pregnant populations, and all clinical translational conclusions remain preliminary until multi-center external validation replicates the model’ s predictive discrimination, calibration, and clinical net benefit. In addition, the high continuous NRI observed (0.862) carries limited standalone interpretive value; this study prioritizes IDI and DCA for judging incremental predictive gain to mitigate interpretive bias associated with NRI metrics.

## Data Availability

The raw data supporting the conclusions of this article will be made available by the authors, without undue reservation.
